# Cohort profile: The Youth and Mental Health Study (YAMHS) – a longitudinal study of the period from adolescence to adulthood

**DOI:** 10.1371/journal.pone.0247036

**Published:** 2021-02-19

**Authors:** Jannike Kaasbøll, Johannes Foss Sigurdson, Norbert Skokauskas, Anne Mari Sund

**Affiliations:** 1 Department of Mental Health, Faculty of Medicine and Health Sciences, Norwegian University of Science and Technology (NTNU), Regional Centre for Child and Youth Mental Health and Child Welfare (RKBU Central Norway), Trondheim, Norway; 2 Department of Health Research, SINTEF Digital, Trondheim, Norway; 3 Department of Child and Adolescent Psychiatry, St. Olav’s Hospital, Trondheim, Norway; University of Sao Paulo Medical School, BRAZIL

## Abstract

The aim of this article is to provide a detailed description of the Youth and Mental Health Study (YAMHS),a population-based, representative (cluster sampling), prospective cohort study that was conducted to investigate risk and resilience factors for mental health conditions, specifically depressive symptoms and disorders, from adolescence to adulthood. The baseline data were collected in 1998 (T1) in two counties in central Norway from 2464 adolescents (response rate 88.3%, mean age 13.7 years). The first follow-up was conducted in 1999 (T2) (n = 2432, response rate of 87.1%, mean age 14.9 years). A subgroup of individuals was assessed at T2 (n = 345) with clinical interviews, and this subgroup was reassessed in 2005 (T3) (n = 265, 70.1%, 20 years). The last follow-up (of participants assessed at T1 and T2) was conducted in 2012 (T4) (n = 1266, 51.9%, 27.2 years). Demographics, depressive symptoms, general psychopathology, suicidal ideation and attempts and psychological and somatic factors were recorded. Among adolescents of both sexes, psychosocial variables were correlated with and predicted depressive symptom severity. The strongest predictors were sex (female), the levels of depressive symptoms the preceding year, and the total number of stressful events. The association between stressful life events and depressive symptoms was moderated by physical activity, while the relationship between stressful events and coping style was mediated by depressive symptoms. The rate of use of specialised mental health services among the depressed was low. The lifetime prevalence of depressive disorders was 23% at 15 years, and the most common disorder was minor depression. Adolescents who attempted suicide were more often victims of violence and less resilient than were non-suicide attempters. The existing longitudinal data from the cohort will be further analysed. Follow-up data will be obtained from existing national registries by links created with individual identification numbers.

## Introduction

Mental health problems in adolescence may persist into adulthood and have lifelong consequences [1–3]. The Youth and Mental Health Study (YAMHS) was designed over 20 years ago to study risk and resilience factors for the development of depressive symptoms and disorders in adolescence. In the preceding decades, a growing understanding had surfaced about the mere existence of depression among children and adolescents [4, 5], and about their ability to report on inner mental states in interviews and on self-reports [6]. At the time, some longitudinal community-based cohort studies had substantially contributed to our understanding of the trajectories of adolescent mental health or, more specifically, depressive symptoms [7] and disorders [8–12], in addition to some seminal clinical studies [13, 14]. At the outset of the present study, major contributing factors for depression had been identified, like the role of lower social class, female gender [15], puberty [16], physical activity [17], family [18], negative life events and stress [19] and cognitive factors [20]. Since then, a range of longitudinal cohort studies on the transition into late adolescence and young adulthood were conducted [21–27]. Nevertheless, very little was known about how these factors interact in early adolescence over to adult age. Prior to the YAMHS, there were no Norwegian longitudinal influential studies of adolescents’ mental health in a population-based cohort including a thoroughly assessed clinically depressed subgroup. With this study design, the results may provide insight useful for recognizing depression as well as testing and improving preventive measures at the community level. In addition, the study may increase our knowledge and educate therapists on how to help young people.

Several factors made this cohort study possible: the stable population in central Norway, the support from school administrations, and the financial support provided by local and national funding agencies. The adolescents were followed up through young adulthood. After the first accumulated data for the project were analysed, distinctive vulnerable groups were identified, meriting additional investigations forming foundation for several PhD theses in the project.

Initially, the YAMHS in central Norway had three specific objectives:

- To study correlates, risk, and protective factors for the development of depressive symptoms in early adolescence.
- To assess the prevalence of, course of, risk factors for and resilience of adolescent depression and suicidality from adolescence to late adolescence.
- To evaluate the short- and long-term effects of bullying in adolescents and young adults.

Over recent years, longitudinal cohort studies have provided invaluable knowledge about continuity and changes in depressive symptoms [28, 29] and disorders [3], investigating both the course and outcomes into adulthood in the community settings. Nevertheless, studies following adolescents including thoroughly assessed depressive cases outside the health system are relatively rare [25, 30–32], and outcome may vary with follow-up time [3].

A limitation of many available studies looking at the adult mental health and psychosocial outcomes of early-onset depression and suicidality in community samples concerns the sole reliance on self-reports [28]. To reduce the influence of potential self-reporting bias (i.e. social desirability and recall-bias), and systematic attrition in longitudinal studies, more objective and comprehensive data are needed [25]. The national registries held by governmental agencies in Norway and other Nordic countries provide extensive resources for register-based epidemiological research [25, 33, 34]. Furthermore, all information in the registers is connected to the unique personal identification number of each citizen, which allows linkage between various registers and population-based cohort studies. The combination of survey and registry data provides extensive information about the individual that is not available when combining different registers alone.

To date, there are few comparable population-based cohorts using linkage to national registers to follow the adolescents into mid-adulthood, exploring the range of depressive problems in relation to risk and resilience factors. One of the exceptions is the Uppsala Longitudinal Adolescent Depression Study (ULADS), a longitudinal cohort study [25] that currently is investigating the overall long-term burden (healthcare costs and earnings) of adolescent (aged 16–17) depression [35, 36]. However, the ULADS completed the follow-up assessments of only depressed adolescents and controls, not the whole cohort. Furthermore, adolescents who self-harm and with suicidal ideation and behaviour are being followed up in a range of studies to young adult [30, 37, 38] and to late adult age [39]. However, these studies bear no clear conclusion on the significance of early adolescence suicidality on later suicidality or functional outcome. To summarize, YAMHS goes beyond existing studies and combines register data with comprehensive self-reports on psychological and somatic strengths and vulnerabilities assessed from early adolescence up to mid-adult age.

The main overall objectives of future investigations pertinent to the YAMHS are to investigate:

- What is the course of depressive symptoms and disorders from early adolescence to mid-adulthood in a general population sample using self-reported data and register data regarding:
  ○ mental health
  ○ physical health
  ○ The use of health services
- What is the course of suicidal ideation, attempts and self-harm from early adolescence to mid-adulthood? What is the significance of suicidality in early adolescence on later mental and physical health?
- How do risk and resilience factors at the individual and environmental level in adolescence and early adulthood affect the above listed outcomes later in adulthood?

The aim of the current cohort profile paper is to provide a comprehensive description of the YAMHS cohort as a research resource for potential collaborations, including an overview of the study population and the collected data, a description of the baseline characteristics and a summary of the main results published up to now.

## Cohort description

### Study population and recruitment process

In 1998, there were 9292 adolescents attending 8^th^ and 9^th^ grades in *Sør- and Nord-Trøndelag* (two counties) in central Norway. The majority (98.5%) of the adolescents attended a public school. A representative sample of this population (2813 students, from 22 schools) was selected with a probability according to the school size (proportional allocation). The exclusion criteria for the schools were as follows: small schools (n = 534), i.e., those without at least one class for each grade level. Twenty-one pupils (0.7%) were not eligible for the following reasons: having been absent from school on the day of the assessment (i.e., were admitted to a hospital, were temporarily located abroad), having been in an institution, having not been proficient in Norwegian, or having recently arrived in Norway. A total of 2792 adolescents were eligible for the study. Both adolescents and their parents received an invitation letter and asking the adolescents to participate in the study; 88.3% of the adolescents and their parents or guardians consented. Thus, the baseline sample population consisted of 2464 adolescents attending the 8th and 9th grades in private and public schools during autumn 1998 (51% females), which had been divided into four strata: 1) the city of Trondheim (n = 484), the suburbs of Trondheim (n = 432), 3) the coastal region of Central Norway (n = 405) and 4) the inland of Central Norway (n = 1143). The mean age at T1 was 13.7 years (range 12.5–15.7, SD 0.6). Data were gathered through questionnaires completed by each individual during two school hours. Teaching personnel were present to clarify questions if needed.

Table 1 displays the key characteristics of the study population at baseline (T1). The study population had a lower proportion of individuals whose two parents were born in a foreign country than did the general population of Norway (2.7%, vs. 5.3%) (Statistics Norway, 1998). A total of 3.9% (n = 95) of the participants were born in a foreign country, which was also lower than the proportion in the general population (5.6%, Statistics Norway, 1998). The non-responders (N = 328) were more often boys and younger adolescents. In the study population, adolescents from the city of Trondheim were less likely to be non-responders than were adolescents from the suburban areas.

**Table 1:** Demographic characteristics of the total sample (N = 2464) at baseline (T1).

	N	%
**Sex**		
Females	1252	50.8
Males	1212	49.2
**Parental SES**		
Professional/executive (upper class)	241	9.8
Upper middle class	704	28.6
Lower middle class	326	13.2
Primary industry	204	8.2
Manual worker	899	36.5
No information/missing	90	3.7
**Ethnicity**		
Both parents Norwegian	2299	93.3
One parent Norwegian	96	3.9
No parents Norwegian	65	2.7
No information	4	0.1
**Living arrangements**		
Living with both parents	1772	71.5
Living with either parent alone	370	15.3
Living with one parent and one stepparent	239	9.8
Sharing time between parents	68	2.8
Other (lived with foster parents or with grandparents)	15	0.6

### Follow-up assessments

The YAMHS consisted of three follow-up assessments of participants aged from 13 years (T1) to 27 years (T4) (Table 2).

**Table 2:** Sample sizes, year of the data collection, response rate, age and sex of the adolescents.

Data waves	T1	T2	T2 subgroup	T3 subgroup	T4
Year	1998	1999	1999/2000	2005	2012
Mean age (years) ^a^	13.7	14.9	15.0	20.0	27.2
Range, age (years)	12.5–15.7	13.7–17.0	13.7–17.0	18.9–21.4	26.0–28.2
N adolescents	2464	2432	345	265 ^d^	1266
% adolescents	88.3% ^b^	87.1% ^b^	94.1% ^c^	76.8%	51.9% ^e^
% girls	50.8%	50.4%	72.5%	76.9%	56.7%

^a^Standard deviation = 0.6 for all waves.

^b^Response rate % at T1 and T2 is calculated based on adolescents invited at T1 (n = 2792 adolescents).

^c^For the subgroup (T2), the response rate is calculated based on a subset (n = 364) at T2 that was invited for a clinical interview using the Kiddie-SADS-PL based on scores on the Mood and Feelings Questionnaire.

^d^Includes interviews and self-reports.

^e^The response rates for adolescents (T4) are calculated based on adolescents participating at T1 and T2 (n = 2532, of which 72 students consented at T2 who had not participated at T1). Note: T1  =  Time 1, T2  =  Time 2 etc.

**Fig 1:**
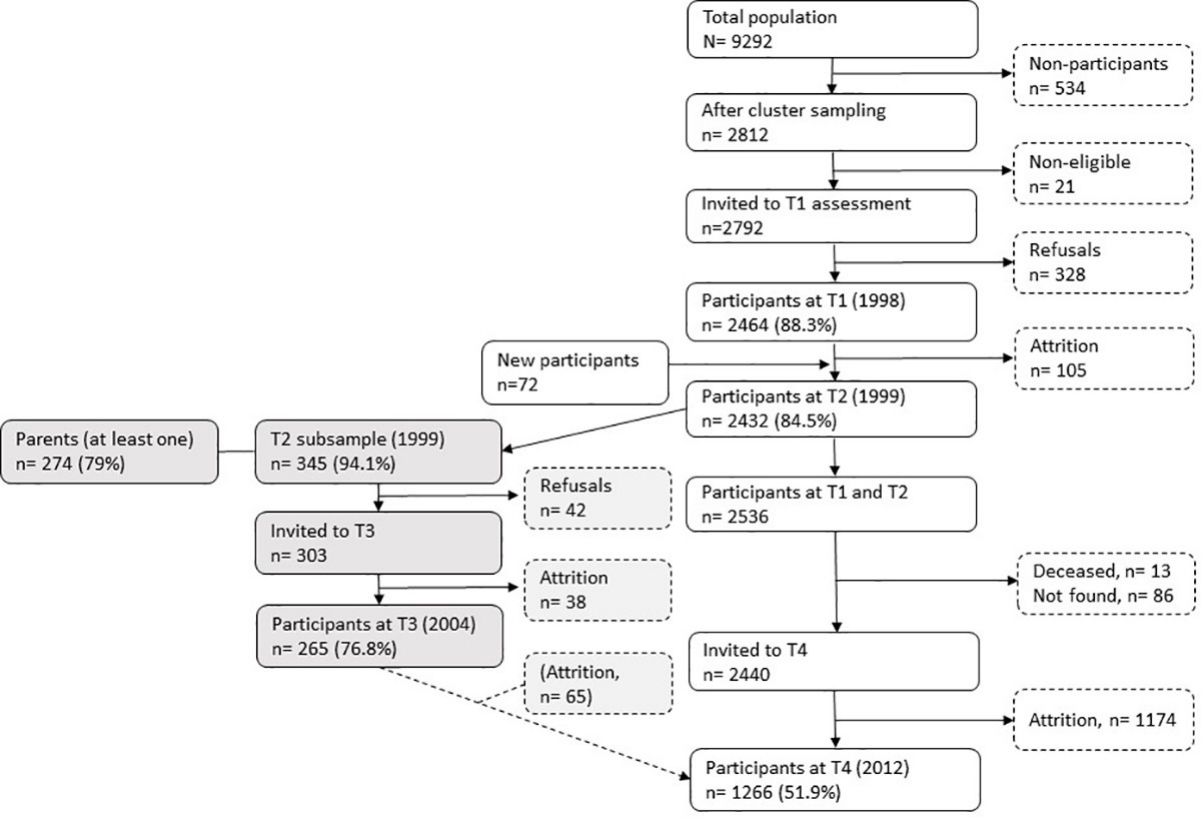
Procedure and flow of participants. Note. T1  =  Time 1, T2  =  Time 2 etc.

The flow of participant selection in the YAMHS is shown in Fig 1. The initial study population was assessed with a questionnaire at two time points (T1 and T2) with a one-year interval (Table 2). T1 was completed on September 15, 1998, and T2 occurred from August 1999 to January 2000, because of intervening interviews. At T2, 2432 adolescents participated with a mean age 14.9 (SD 0.6). Furthermore, at T2 in 1999, a case-control sub-group study (N = 345) on age- and sex-matched scores of depressive symptoms on the Mood and Feelings Questionnaire (MFQ) [40] was conducted. All high scorers (MFQ> = 26) and a sample of low/middle scorers (0-6/7-25), were invited for an interview using the Kiddie Schedule for Affective Disorders and Schizophrenia (present and lifetime version) [41]. For 79% of the interviewed participants, at least one parent participated in separate interviews. The parents also filled in self-report questionnaires. Five years later, in 2005, this subgroup of adolescents was reassessed (T3) using the same interview (telephone interview, N = 242) in addition to a self-report questionnaire (N = 252). Individuals who were assessed at T1 or T2 were invited to participate in a digitalized follow-up survey in spring 2012 (T4).

### Attrition

Of the individuals who had participated at T1, one hundred and five pupils (4.3%) did not participate one year later (T2). The reasons for non-participation at T2 were relocation, illness, refusal to participate, a serious disability, and school leave. The non-participants were characterized as having higher depressive symptom scores at T1 and being more likely to have a non-Norwegian background. No sex-, grade- or SES-related differences between the non-participants and the participants were found. At T4, 96 participants could not participate because they had died (n = 13) or they did not have an identifiable home address (n = 83); thus, 2440 participants were invited to participate in this follow-up investigation, of whom 1266 (51.9%) participated (see the flowchart in Fig 1). The responders at T4 were more commonly women than were the non-responders, and fewer responders had a non-Norwegian ethnicity. The upper middle class was overrepresented among the parents of the responders, whereas workers were underrepresented.

### Measurements

Identical self-report measures were used at all four earlier assessment-points with modifications and additional questions related to the life phase. Parental information was gathered at T2. Information about mental and physical health, including self- harm and suicidality, use of health services, in addition to life-style factors and adaptive functioning was collected. Also, assessment of individual factors as pubertal timing, psychological coping styles, handling of stress and levels of physical activity was made. Factors in the immediate and more distant environment like chronic and acute life stress, including bullying, relationships to parents, partners and friends, family functioning, parental health, school/job factors, SES, and ethnicity were included. The measures were valid and reliable, commonly used in population and clinical studies on mental health, some in a shortened version. For some purposes questions and scales were made for the study. For example, the EASQ (Early Stress Questionnaire Scale [42]) was compiled by using existing scales, knowledge from health personnel, and piloting it in the classroom to make it relevant for this age group at the time. Coping with depression scale was correspondingly constructed based on earlier research and on coping [43] and rumination theory [44].

### Main outcome variables in the study

- The Kiddie-SADS-PL (Schedule for Affective Disorders and Schizophrenia for school aged children (Present and Life-time version) [41] a semi-structured interview covering DSM-IV diagnoses with an introduction covering developmental and somatic history. Assessed at T2 (parents included) and T3.
- The Mood and Feelings Questionnaire (MFQ) [40] is a 34 items questionnaire assessing depressive symptoms and covering DSM-IV criteria of major depressive disorders at all time-points.
- The ASEBA system: CBCL (parents T2) [45], YSR (Youth Self-Report) [46] and ASR (Adult-Self-Report) [47] is a broad instrument covering functioning in major areas, including anxiety and depressive symptoms and conduct problems at all time-points.
- The Adult Attachment Interview (AAI) [48] at T3.

Details on the measures and respondents at each time point are presented in Table 3.

**Table 3:** Instruments in the “Youth and Mental Health Study”.

		Study waves and respondents (Y = youth and P = parents)				
		T1	T2	T2 sub^a^	T2 sub^a^	T4
**Sociodemographic variables **						
Sociodemographic data I	Sex, age, nationality, i.e. parents’ country of origin, years of residence in Norway, adoption status. Socio-economic status (SES)—parental occupation (ISCO-88) [49]	Y	Y	Y P	Y	Y
Sociodemographic data II	Living situation, economy				Y	Y
	Education /occupation					
	Own children					
**Mental health **						
	Kiddie-SADS- PL [41]			Y P		
ASEBA: Internalizing and externalizing problems	ASEBA, YSR [46]/ASR [47]	Y	Y	Y P	Y	Y
Depressive symptoms	The Mood and Feelings Questionnaire (MFQ) [40]	Y	Y		Y	Y
Coping	Coping with depression scale^b^ based on Response Style Questionnaire [50]	Y	Y		Y	Y
Anxiety symptoms	Beck Anxiety Inventory [51]				Y	
Resilience	Connor-Davidson Resilience Scale (CD-RISC) [52]				Y	
Metacognitive Questionnaire	Covering metacognitive beliefs [53]					Y
Cognitions	Dysfunctional Attitude Scale, short version [54]	Y	Y		Y	Y
Stressful life events	The Early Adolescence Stress Questionnaire” (EASQ) ^b^	Y	Y		Y	Y
Daily hassles	The Daily Hassels scale [54]	Y	Y		Y	Y
Coping with stress	Coping Inventory for stressful situations [55]	Y	Y		Y	Y
Self-perception	Self-Perception Profile for Adolescents (SPPA) [56]	Y	Y		Y	Y
Self-consciousness	The Self-consciousness scale [54, 57]	Y	Y		Y	
Sex role	Bem Sex-role Inventory Short form [58]	Y	Y			
Functional impairment	Psychiatric problems^b^	Y	Y	Y P	Y	Y
**Use of services**	For mental health problems^b^	Y	Y	Y P	Y	Y
**Relationships**						
Family	McMaster Family Adjustment Device [59]	Y	Y	Y P	Y	
Conflict with parents	Selected items [54, 60]	Y	Y	Y P	Y	
IPPA	Inventory of Parent and Peer Attachment Scale [61]					
Relationships to own children, friends, partner	ASEBA [45–47]	Y	Y	Y P	Y	Y
**Suicidal thoughts/act **						
Ideation, self-harm, suicidal attempts, hopelessness	[54, 62, 63]	Y	Y		Y	Y
Suicidality	Suicidal Intention Scale [64]			Y P		
Bullying	Type, frequency, duration [65]	Y	Y			Y
**Somatic variables **						
Pubertal stage	Pubertal Development Scale [66]	Y	Y			
BMI	Body Mass Index (weight/height^2^)	Y	Y		Y	Y
Somatic illnesses	ICPC- criteria (International Classification of Primary Care [67] Medication^b^	Y	Y	Y P	Y	Y
General health	One question [68]	Y	Y		Y	Y
Pain	Regular and troublesome pain complaints; frequency and duration of pain; headache and migraine^b^	Y	Y		Y	Y
Eating habits	^b^	Y	Y		Y	
Physical Activity	Items modified after Paffenbarger et al. [69]	Y	Y		Y	Y
Sleep	From ASEBA and DSM-IV [70] s	Y	Y		Y	Y
Substance use	Smoking, alcohol, narcotics*	Y	Y	Y P	Y	Y
Physical impairment	Reduction activity last year*	Y	Y		Y	Y
**School variables**						
Adaptation	^b^	Y	Y	P	Y	Y
Dyslexia	^b^	Y	Y		Y	Y
Grades	^b^ Items modified based on ASEBA (YSR and CBCL)	Y	Y	P		
**Parental Variables**						
ASEBA	CBCL [45]			P		
Parental Depressive symptoms	Beck Depression Inventory [71]			P		
Relationship with child	Parental Bonding Inventory [72]			P		
Internalizing/externalizing problems ASEBA	Adult Self Report [47]			P		
EASQ	Early Adolescent Stress Questionnaire^b^			P		
Interview	Child developmental, somatic and psychiatric history. Alcohol use, Somatic Health Depressive disorders DSM IV [70], Family history of psychiatric disorders			P		
	Perceived economic satisfaction					
	Physical illness or disability^b^					

Note: T1  =  Time 1, T2  =  Time 2 etc.

^a^Sub = Subgroup.

^b^Items/scales made in the project were mainly adapted from existing scales and previous research.

### National registers

Individual record-linkages between the YAMHS and the different registries outlined in Table 4 will be constructed by means of the unique personal identifier assigned to all Norwegian residents, Consent from the participants to link data to registers were retrieved from all participants who participated at wave T4. Regarding health care utilisation, information will be retrieved from the Primary Health Care Database (KUHR) and the Norwegian Patient Registry (NPR), the first including data for primary care physicians and other health care professionals eligible for reimbursement from the National Insurance Scheme, and the latter covering both inpatient and outpatient treatment in the specialist health care services. These databases include consultation dates and diagnostic information according to the ICPC-2 and the ICD-10 coding systems [33, 73]. Relevant socioeconomic and demographic factors will be included to control for potential confounding, provided by the registries of Statistics Norway. The National Welfare Database (FD-Trygd) include information regarding employment and sickness absence histories, as well as diagnoses for individuals granted disability pension. Examples of variables to be used from the registries is shown in Table 4.

**Table 4:** Relevant data sources–national registry data.

Relevant data sources	Available since (year)	Examples of variables	Variable of interest
The Specialist Health Care Database (NPR)	2008	Date of admission and Discharge, ICD-10 diagnosis code, Wait list data	Outcome
The Norwegian Prescription Database (NorPD)	2004	Describe drug use patterns	Outcome
The National Education Database (NUDB)	1970	Educational attainment, School grades/absence	Confounder
The National Welfare Database (FD-Trygd)	1993	Use of welfare services, Employment, Income, Benefit diagnoses	Outcome/Confounder
The Primary Health Care Database (KUHR)	2006	Date of consultation, ICPC-2 diagnosis code	Outcome
The Causes of Death Registry	2001	Causes of Death	Outcome

### Patient and public involvement

Patients were not involved. Public representatives (i.e., schools) were involved in preparing (e.g., piloting the questionnaires) and conducting the study. In the first wave (T1) and fourth wave (T4), age-appropriate focus groups were involved in the design of survey questions. Information letters containing results and publications were distributed to all respondents in all waves.

### Ethics approval

The “Youth and Mental Health Study” project has been approved by REK (Regional Committee for Medical Research Ethics) in all earlier waves (Latest approval: REK midt: 2011/1454 (T4)). Prior to all study waves have been conducted, all eligible participants (and the parents or legal guardians) received a letter by post informing about the project and an invitation to participation. At wave T4 (year 2012, mean age 27.2 years), the letter of information and invitation to participate was extended to include a consent to link data to the central national registers in future studies. For the use and linkage of the registers, the project will seek approval by the Norwegian Data inspectorate and the register owners: e.g. the Norwegian Board of Health, Statistics Norway and The Norwegian Directorate for Education and Training. In addition, this study will perform Data Protection Impact Assessment (DPIA), as it is now required under the GDPR (General Data Protection Regulation) for any processes that could put a data subject’s privacy at risk.

## Findings to date

### Prevalence, correlates and risk factors for depressive symptoms in early adolescence

For the 90^th^ percentile score on the MFQ (MFQ > = 25), 8.6% of the participants were high scorers, more girls than boys (12.6% vs 4.4%). Concentration difficulties most strongly predicted a high depression score [74]. A range of correlates with depressive symptoms were identified, including experiencing daily hassles (daily stress), stressful life events last 12 months, school-related stress, frequent/multiple occurrences of pain, and reading difficulties and having few friends, a low level of vigorous exercise, a non-Norwegian ethnicity, and non-working mothers. For girls, having an older age, many siblings, an advanced pubertal stage and a high BMI (body mass index) were associated with depressive symptoms [75]. In the longitudinal analyses, the following variables were potential risk factors: the levels of depressive symptoms in the preceding year, the female sex, an insecure attachment to parents (but not to friends), the occurrence of stressful life events, a low level of vigorous exercise, and frequent headache and limb pain. The female sex and stress were the most significant risk factors [76, 77]. Except for a sedentary lifestyle, which emerged as a risk factor for boys only, and having a non-Norwegian background and experiencing school-related stress, which were risk factors among girls only [78], the risk factors were strikingly similar between the two sexes. The moderating effect of vigorous exercise on stress in relation to the development of depressive symptoms [76] and the moderating effect of special education on the association between reading difficulties and mental health problems are noteworthy [79].

Headache was found to be associated with and to be a precursor of depressive symptoms [80, 81]. Female sex, greater psychosocial impairment (i.e., impairment during leisure-time activities or seeing friends because of disease, injury, or pain), and comorbid depressive symptoms were indicators for both the short- and long-term prognoses of frequent headaches, illustrating the bidirectional associations between somatic symptoms, e.g., pain and emotional symptoms [82].

### The prevalence and course of adolescent depression and parental risk factors

According to the data from the sub-group of mainly high scorers (depression), almost one in four subjects had lifetime depression according to population estimates. The point prevalence rates at 15 years of major depressive disorder (MDD), dysthymia and double depression (MDD and dysthymia) were 2.6%, 1.0% and 0.6%, respectively, for depressive disorder not otherwise specified (NOS) 6.3%. All disorders were characterized by a long duration of episodes with large variability and dysthymia onset before 12 years of age [83]. The prevalence of comorbidities with other disorders was high, i.e., 60% at 15 years and 52% at 20 years (unpublished data). MDD and dysthymia were strongly associated with the female sex and not living with either biological parent. For depressive disorder NOS, no sex differences were found [83]. The following four distinctive trajectories from T2 to T3 were identified: not depressed, becoming depressed, continuously depressed and improving from ages 15–20. There was considerable stability overall, with no sex differences in patterns across age groups [84]. Maternal and paternal internalizing symptoms as well as less secure attachment were associated with the development of clinical depression in adolescents from ages 15–20 years [85].

### Associated and predictive factors of attempted suicide among depressed adolescents

The rates of self-harm and suicidal attempts (SA) at T1 were low, 2.9% and 3.0%, respectively, with girls being in preponderance. Being a girl and the level of depressive symptoms the year before were the strongest predictors for incident self-harming behaviour a year later [86]. For the sub-group of adolescents (T1, T2 and T3) [87], the results showed that the main risk factors for SA from 15 to 20 years were having a history of SA, experiencing a family divorce, having depressive disorders, having specific cognitive depressive symptoms and being a victim of violent events. High levels of resilience protected against SA [88]. Among individuals who attempted suicide, antecedent depression predicted decreased task-oriented coping and increased emotional coping at 20 years of age. Consistently, repeaters reported having more stressful events, more severe depression and lower levels of task-oriented coping [89, 90].

### Short- and long-term effects of bullying in adolescence

Four mutually exclusive groups in the study population assessed at T1 and T2 were distinguished: bullies, victims of bullying, individuals who were aggressive towards others, and individuals who were not involved (used as a comparison group). Both being bullied and being aggressive towards others were associated with a high level of depressive symptoms [91]. Being bullied predicted the occurrence of suicidal ideation one year later [92]. The association between being bullied and depressive symptoms in adolescence was both moderated and partially mediated by emotional coping [93]. All groups involved in bullying were more likely to have a poorer outcomes related to psychosocial functioning and mental health at 27 years of age [94], including more externalizing and internalizing problems and a higher risk for hospitalization than the non-involved group. Victims of bullying specifically had an increased risk for depressive problems and reduced levels of psychosocial functioning and were more likely to seek help for mental health problems [95]. Positive coping strategies in adolescence did not moderate these relationships. Last, being bullied in adolescence strongly predicted SA and self-harm [96].

### Sex differences

The girls had higher scores for depressive symptoms than did the boys at all time points [76]. However, the scores of the girls decreased from adolescence to adulthood, whereas the scores of the boys remained constant (unpublished data). The coping strategies used and their associations with stressful events differed by sex. The association among emotional coping, school stress and network stress (friends) was stronger among girls than among boys, while the association between avoidant coping and major family events was stronger for boys. For task coping, no sex differences were found. Depressive symptoms mediated the relationships between stressful events and psychological coping style [97]. For both sexes, the level of event-related stress the year before predicted an increase in depressive symptoms one year later, although the areas of stress differed. The prevalence of severe depression (MDD and dysthymia) was higher among the girls, especially those who had a more severe course of depression, than among the boys, while for minor depression (depression NOS), there were no differences between sexes. The majority of the sub-group of mainly depressed adolescents was girls (72.5%); hence, the risk factors found to be related to suicidal ideation, self-harm and SA can be generalized only to girls. The boys who were bullied during adolescence were the most vulnerable group and reported the highest levels of lifetime SA in adulthood, while formerly bullied adult women had the highest risk of self-harm [96].

### Use of health services and prevention

Among the individuals who were clinically depressed at 15 years of age, an estimated population share of 50% had received mental health care in their life-time, but seldom from the specialised services and the affective symptoms were rarely reason for contact [83]. Individuals who were bullied during adolescence, not unexpectedly, had a high rate of use of mental health services. This result has also been reported for adults [95]. Individuals who attempted suicide were more likely to have been in contact with child protection services than with mental health services in adolescence [98]. In conclusion, detecting, preventing and treating depression and preventing bullying in early adolescence has the potential to improve both psychosocial functions and mental health both short- and long-term. Promoting positive coping and improving relationships with parents is potentially protective during adolescence. Stressful events, especially those related to being a victim of violence, represent a strong risk factor for suicidality among depressed young people and should be remedied and prevented.

### Publications

To date, 30 peer-reviewed papers, five doctoral theses [89, 99–102] and several masters’ theses that include the YAMHS data have been completed. In addition, one doctoral thesis compared the population data from this study with a clinical population [103]. A full list of YAMHS publications is available at https://www.ntnu.no/rkbu/ungdom-og-psykisk-helse

## Strengths and limitations

The strengths of the YAMHS are that it 1) includes a relatively large school-based representative study population with a high response rate, 2) has a long follow-up period of 12 years from adolescence to adulthood, and 3) is a subgroup study, with the over-inclusion of adolescents who reported high levels of depressive symptoms and were assessed for depressive disorders. An interview that was conducted 5 years later provided valuable information on resilience and risk factors for the development of depressive disorders and suicidality during the late adolescent period.

This study included parents, and it is one of the few studies that has included fathers. By using the Adult Self-Report (ASEBA) at T2 subgroup, we obtained a broad dimensional assessment of parental mental health. Additionally, by conducting a clinical interview, we assessed general somatic health, minor and major depression and alcohol use among parents. Nevertheless, a broader clinical interview would have offered a deeper perspective on parental mental health as a putative risk factor for adolescent depression.

This study investigated a 12-year time span, which corresponds to a long developmental period marked by substantial maturation and changes in individuals. A weakness of the study is therefore that the relatively large time period spans assessment points after early adolescence. Intervening factors could have changed trajectories and affected individuals’ mental health in unforeseen ways that were not accounted for in this study. Furthermore, although the response rate was excellent at both T1 and T2, it was moderate at T4. Attrition analyses showed that there were small differences between the responders and non-responders at T4 regarding sex, parental SES, and ethnicity. This discrepancy can potentially lead to the over- or underestimation of the results. However, the study population assessed at T4 is large and heterogeneous in terms of sex and demographic markers, which suggests that it is possible to generalize the study findings to the target population.

Except for the sub-group results for T2, all results in the study were determined based on adolescent and young adult self-reports. Information from teachers and more objective measures regarding behaviour, somatic status, physical activity, sleeping patterns, etc. would have strengthened the validity of the findings. Biological material was not included in this study, which has prevented us from conducting both hormonal analyses as well as genetic sampling; genetic sampling can be useful to study genetic vulnerability and test genetic risk profiles in this relatively large study population. With the subgroups including adolescents with a depressive disorder and controls followed from adolescence, we are in a good position to be able to study the unfolding of depressive disorders in young people, which is not always detected by health services. Still, since our study also includes parental data, an environmental versus a heredity perspective can be applied.

### Future plans

**Fig 2:**
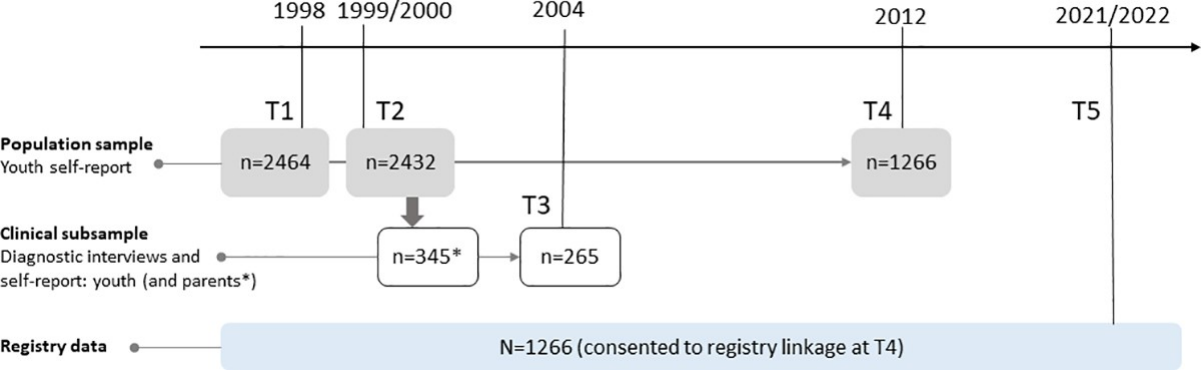
Future plans: Linkage of the Youth and Mental Health Study with national registry data.

We plan to explore our findings further and link the data to the different national registers that are of high quality (Fig 2). The YAMHS participants will be around 36 years at the time of entering the register study. The YAMHS project group has in collaboration with the Child and adolescent mental health services (BUP) at St. Olav’s University Hospital recently received a full time 3 years postdoctoral research fellowship grant by the Liaison Committee’s (Samarbeidsorganet), Helse Midt-Norge RHF (November 2020). The title of the postdoctoral project is “A longitudinal register linkage study from adolescence to adulthood: risk, resilience, and youth depression outcomes”. We also plan to establish extended collaboration with researchers working with similar datasets and to explore the possibilities of collaborating with the researchers conducting the HUNT study [104], which, over decades, has documented the health of the general population in Nord-Trøndelag, the same geographical area in which half of the participants in the YAMHS lived.

### Collaboration

Researchers who are interested in future collaborations using the YAMHS database should contact the research group at yamhs@rkbu.ntnu.no. The study is designed to be a limited access resource in which external researchers can access the data when we have available resources to facilitate and administer collaboration, rather than an open access resource. Researchers affiliated with a qualified research institution are encouraged to send a brief proposal to the group. The YAMHS has a website with updated contact information: https://www.ntnu.no/rkbu/ungdom-og-psykisk-helse.
